# Derivation of an updated brief multivariable prediction model to detect panic-related anxiety in emergency department patients with cardiopulmonary complaints

**DOI:** 10.3389/fpsyt.2026.1750468

**Published:** 2026-02-11

**Authors:** Sharon C. Sung, Felicia J.L. Ang, Arul Earnest, Leslie E.C. Lim, Shreshtha Jolly, Gilaine Ng, A. John Rush, Marcus E.H. Ong

**Affiliations:** 1 Programme in Health Services Research & Population Health, Duke-NUS Medical School, Singapore, Singapore; 2 School of Public Health and Preventive Medicine, Monash University, Melbourne, VIC, Australia; 3 Department of Psychiatry, Singapore General Hospital, Singapore, Singapore; 4 Bloomberg School of Public Health, Johns Hopkins University, Baltimore, MD, United States; 5 School of Social Sciences, Singapore Management University, Singapore, Singapore; 6 Curbstone Consultant LLC, Dallas, TX, United States; 7 Duke-NUS Medical School, National University of Singapore, Singapore, Singapore; 8 Department of Emergency Medicine, Singapore General Hospital, Singapore, Singapore

**Keywords:** emergency department, panic attack, panic disorder, prediction model, screening interview

## Abstract

**Background:**

Patients with panic related-anxiety (i.e., panic attacks or panic disorder) frequently present to emergency departments (EDs) with cardiopulmonary complaints but are often undiagnosed, which can lead to recurrent visits and prolonged distress. This study aimed to derive a new symptom-based multivariable diagnostic prediction model to detect panic-related anxiety in ED patients with cardiopulmonary symptoms.

**Methods:**

We conducted a single-blind prospective derivation study over 15 months in the ED of a major tertiary hospital in Singapore. Patients presenting with symptoms of palpitations, chest pain, dizziness, or difficulty breathing were assessed using the Structured Clinical Interview for DSM Disorders (SCID) to diagnose panic-related anxiety. A stepwise multivariable prediction model was constructed using 13 SCID-defined panic symptoms as predictors, with the diagnosis of panic-related anxiety as the outcome. Diagnostic accuracy was evaluated through sensitivity, specificity, receiver operating characteristics (ROC), and the Youden index.

**Results:**

321 eligible patients were included, with 39% meeting criteria for panic-related anxiety. The optimal cutoff (≥3 symptoms) in the derived model achieved an area under the curve (AUC) of 0.88, sensitivity of 78.4%, specificity of 85.7%, a Youden index of 64.1%, classified 82.9% correctly, positive likelihood ratio=5.4880, and negative likelihood ratio=0.2520.

**Conclusions:**

This newly derived model demonstrated strong diagnostic accuracy in identifying panic-related anxiety among ED patients with cardiopulmonary complaints, suggesting its potential utility in clinical screening. Implementation of this model may facilitate timely diagnosis, reduce repeated ED visits, and improve patient outcomes.

## 1 Introduction

Patients with panic-related anxiety (i.e., panic attacks or panic disorder) use primary healthcare services more frequently than the general population (1, 2) and generate the highest use of emergency department (ED) services among patients with anxiety disorders (3). Even when recognized and treated, panic disorder is often chronic and recurrent, contributing to repeated healthcare use over time. These patients frequently present to the ED with cardiopulmonary complaints such as palpitations, chest pain, dizziness, and shortness of breath, mimicking symptoms of cardiopulmonary emergencies (i.e. myocardial infarction, acute bronchoconstriction) (4). Indeed, panic attacks account for a substantial proportion of ED visits for non-cardiac chest pain, and longitudinal studies show that approximately 70–80% of patients diagnosed with panic disorder in the ED experience recurrent ED visits (5, 6). This symptom overlap often complicates timely diagnosis, leading to repeated ED visits, increased healthcare costs, and poorer long-term outcomes for these patients (1, 2, 7).

Despite high rates of ED presentation among individuals with panic-related anxiety, diagnosis is frequently missed at discharge (6, 8, 9), with many patients left untreated for their underlying anxiety condition. Consequently, there is a need to improve the ability of ED personnel to efficiently identify panic-related anxiety among ED patients. Our team previously developed an initial multivariable prediction model consisting of seven common panic attack symptoms (palpitations, derealization, paresthesia, shortness of breath, chills or hot flushes, dizziness, and fear of losing control or going crazy). This brief interview demonstrated potential in accurately identifying panic-related anxiety in patients with cardiopulmonary complaints, successfully identified panic-related anxiety in a sample of 200 adult ED patients with cardiopulmonary complaints at an accuracy rate of 85% (area under the curve (AUC) = 0.90, sensitivity = 82%, specificity = 88%) (8).

Our objective in the current study is to derive an improved predictive model that would offer more robust diagnostic accuracy in identifying panic-related anxiety among ED patients. By improving screening accuracy, we aim to support ED clinicians in making faster, more accurate diagnoses, thereby reducing the risk of misdiagnosis, decreasing repeat ED visits, and ensuring that patients receive appropriate treatment and follow-up care for anxiety-related conditions.

## 2 Materials and methods

### 2.1 Study design

This single-blind, prospective derivation study was conducted over a 15-month period in the ED of a major tertiary care hospital in Singapore. This study followed the Transparent Reporting of a multivariable prediction model for Individual Prognosis or Diagnosis (TRIPOD) guidelines for derivation studies.

### 2.2 Study setting

Participants were recruited between 8:00 a.m. and 6:00 p.m., Monday through Friday, from a high-volume ED serving approximately 200,000 patients annually in Singapore. As an ethnically and linguistically diverse city-state located in Southeast Asia, this setting provided a diverse patient population with varied linguistic and ethnic backgrounds. Singapore has a resident population of 4.04 million, made up of ethically Chinese (74.3%), Malay (13.5%), Indian (9%), and other ethnic groups (3.2%) (10). Over 97% of the population is literate in one (25.7%) or more than one (74.3%) languages. The most common first language spoken by residents is English (48.3%) followed by Mandarin Chinese (29.9%) (10).

### 2.3 Participants

Eligible participants were English or Mandarin-speaking patients with mild to moderate symptoms on the Patient Acuity Category Scale (11) who were not in danger of acute collapse or in need of resuscitation, aged at least 21 years (the legal age of consent in Singapore), with a chief complaint of palpitations, chest pain, dizziness or difficulty breathing. These symptoms often overlap with both panic-related anxiety and more acute cardiopulmonary conditions, making them critical for the model’s screening utility.

Patients allocated to the most severe triage category were excluded due to the need for acute management. Police cases, patients who were unwilling or unable to complete study procedures, and those who presented with altered mental status, dementia, or psychosis were also excluded.

### 2.4 Procedures

Upon triage, clinical research coordinators identified potential participants through chief complaints documented in the electronic medical records. Following an initial assessment by the ED physician to ensure clinical stability, coordinators provided study details to patients and obtained written informed consent prior to initiating study procedures. All procedures were approved by the hospital’s Institutional Review Board.

Following consent, participants underwent a structured diagnostic interview to establish the diagnosis of panic disorder and the presence of panic attacks using a modified version of the Panic Disorder Module the *Structured Clinical Interview for DSM Disorders* (SCID) (12). The SCID is a well-validated semi-structured interview used to diagnose lifetime and current (past month) Axis I psychiatric disorders. For the present study, the SCID was modified to assess all 13 panic attack symptoms. Instead of ending the interview if the participant answered no to the first screening item (“Have you ever had a panic attack, when you suddenly felt frightened, or anxious or suddenly developed a lot of physical symptoms?”), the interviewer proceeded to assess the presence or absence of each of the panic attack symptoms individually. This modification was made to address the fact that ED patients frequently present early in the course of illness with non-fearful panic attacks (13). Hence they may not be aware that their symptoms panic-related when they first present to the ED.

The SCID Panic Disorder module was administered by trained bilingual clinical interviewers fluent in English and Mandarin following a rigorous forward and back translation process, using standardized translated prompts to ensure conceptual consistency across languages. The interviewers administered the SCID and documented each symptom as either present or absent (scored 1 or 0), in addition to coding a diagnosis of panic attack(s), panic disorder, or no panic-related anxiety. All interviewers were blind to the clinical diagnosis provided by the ED physician, which was obtained from the medical record after the SCID diagnostic interview. Inter-rater reliability was high for panic disorder (κ=0.82) and panic attacks (κ=1.00). Weekly consensus meetings were held throughout the study period. Diagnostic uncertainties were adjudicated by the study psychiatrist (LL).

Participants also completed a the Clinical Report Form (CRF) which captured patient-reported demographics, lifetime medical history, frequency of ED visits and hospital admissions in the past year, recent life events in the past year, physician diagnosis for the current ED visit, and discharge disposition.

### 2.5 Sample size determination

*A priori* sample size calculations indicated that a total sample size of 310 participants would be sufficient to estimate the area under the curve at 0.85 with a 95% confidence interval within a reasonable width of 0.125, assuming that approximately 20% of participants met criteria for panic-related anxiety.

### 2.6 Model derivation

The procedures and results to develop the original model are detailed in Sung et al. (8). To derive our updated predictive model, we used the SCID symptom checklist as the primary predictor set, with presence/absence of panic-related anxiety diagnosis (as diagnosed by the SCID) as the outcome variable. We entered all 13 panic attack symptoms into a stepwise multivariable logistic regression with an entry probability of 0.01 and an exit probability of 0.05 to identify symptoms that independently predicted a panic-related anxiety diagnosis.

### 2.7 Performance of updated model

Model performance was assessed by calculating the area under the receiver operating characteristic (ROC) curve, sensitivity, specificity, positive and negative likelihood ratios, and the Youden index. We used Youden Index for the best balance of sensitivity and specificity. We evaluated various cutoff points for panic symptom counts to identify the optimal threshold for the updated model’s clinical application. All analyses were conducted using Stata, and confidence intervals were calculated for all diagnostic performance metrics.

## 3 Results

### 3.1 Participants

**Figure 1:**
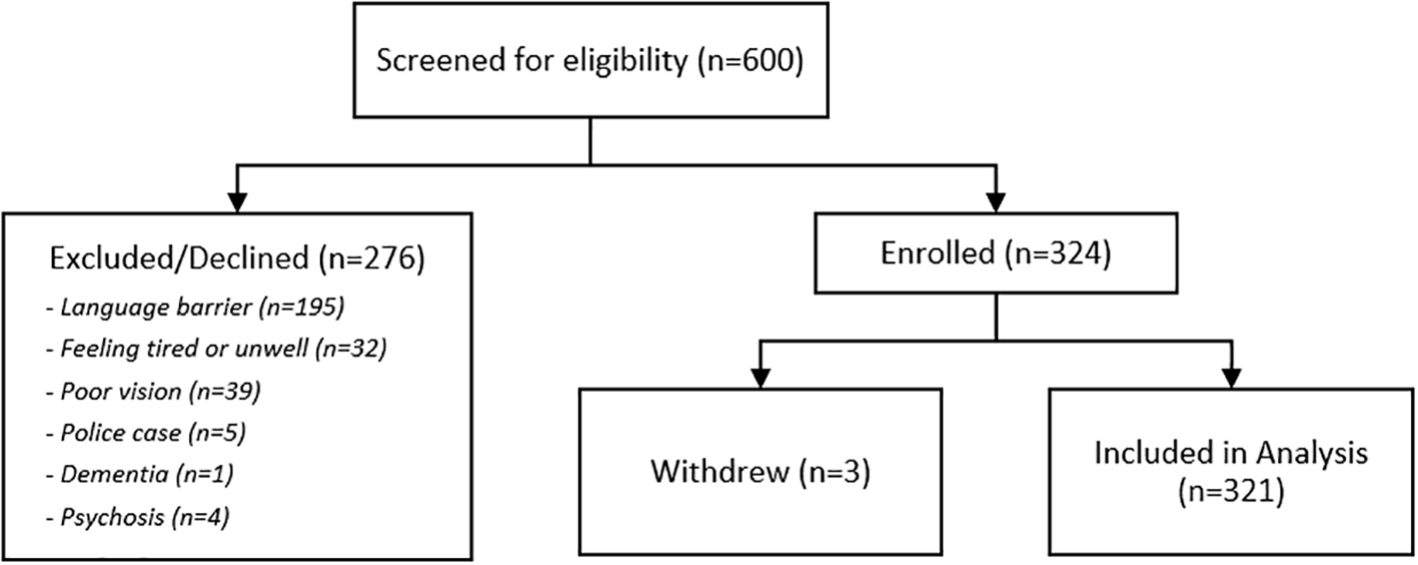
Participant flow diagram.

We recruited 324 eligible participants for the study (See **Figure 1**). The most common reason for exclusion was lack of proficiency in the two study languages. Three participants withdrew before completing all study procedures, resulting in a final sample of 321 participants. Full details of sample demographics are shown in **Table 1**. The mean age was 52.3 years (SD = 12.4), with a higher proportion of males (68.9%) than females. The sample was ethnically diverse, with 61.4% Chinese, 13.7% Malay, 19.3% Indian, and 5.6% representing other ethnic groups. The majority of participants presented with a chief complaint of chest pain (92.2%), followed by dizziness (5.0%) and palpitations (2.8%).

**Table 1:** Sample demographics and clinical features.

Demographic & Clinical Features	External validation sample n=321
Age in years (mean ± SD)	52.3 ± 12.4
Gender, n(%)	
Male	221(68.9)
Female	100(31.1)
Marital status, n(%)	
Single	43(13.4)
Married/living with partner	249(77.6)
Divorced/separated/widowed	29(9.0)
Race/ethnicity, n(%)	
Chinese	197(61.4)
Malay	44(13.7)
Indian	62(19.3)
Eurasian	2(0.6)
Other race/ethnicity	16(5.0)
Education, n(%)	
Primary or below	35(10.9)
Secondary/Vocational	155(48.3)
Pre-university/Polytechnic	68(21.2)
University/Postgraduate	63(19.6)
Occupational status, n(%)	
Managerial/Professional	82(25.6)
Executive/Sales	35(10.9)
Clerical/Technical	44(13.7)
Self-employed	29(9.03)
Retired	29(9.03)
Not working	17(5.30)
Others	85(26.5)
SCID diagnosis, n(%)	
Panic attack(s)	93(29.0)
Panic disorder	32(10.0)
Primary discharge diagnosis, n(%)	
Chest pain	282(87.9)
Angina or myocardial infarction	7(2.2)
Dizziness and giddiness	16(5.0)
Palpitations	9(2.8)
General symptoms	3(0.9)
Other ICD diagnosis	3(0.9)
Anxiety state*	3(0.9)
ED referral, n(%)	
Psychiatry	2(0.6)
Cardiology	190(59.2)
Respiratory medicine	7(2.2)
Gastroenterology	8(2.5)
Internal medicine	10(3.1)
Other specialist outpatient clinic(s)	8(2.5)

Using the SCID, 39% (n=125) of participants met diagnostic criteria for panic-related anxiety, either as isolated panic attacks or as part of a panic disorder diagnosis. Among those diagnosed, the most common symptoms reported included shortness of breath, dizziness, and palpitations, which were frequently co-occurring with chest pain. Fewer than 1% of participants received an anxiety-related diagnosis upon ED discharge, and only 0.6% were referred to outpatient psychiatry for follow-up, highlighting the discrepancy between clinical presentation and discharge diagnosis (see **Table 1**).

### 3.2 Derivation of updated model and performance

As presented in **Table 2**, the original model did not perform as well in the external validation sample. When applying the same cutoff score of ≥ 3 in the external validation sample, the original model’s performance declined, with a classification accuracy of 77.9%, reduced sensitivity of 60%, and a Youden index slightly below 50%.

**Table 2:** Performance of the original and updated prediction models in the external validation sample.

Original prediction model						
Panic symptoms: Paresthesia, Derealization or depersonalization, Chills or flushes, Fear of losing control or going crazy, *Shortness of breath, Dizziness, Palpitations						
Score	Sensitivity	Specificity	Classified Correctly	LR+	LR-	Youden Index
≥ 1	98.40%	34.70%	59.50%	1.5068	0.0461	33.10%
**≥ 2**	**86.40%**	**65.30%**	**73.50%**	**2.4904**	**0.2082**	**51.70%**
**≥ 3**	**60.00%**	**89.30%**	**77.90%**	**5.6**	**0.448**	**49.30%**
≥ 4	36.80%	93.90%	71.70%	6.0107	0.6732	30.70%
≥ 5	22.40%	98.50%	68.90%	14.6347	0.7881	20.90%
≥ 6	6.40%	99.50%	63.20%	12.5441	0.9408	5.90%
≥ 7	0.80%	100%	61.30%	-{{-}}-	0.992	0.80%

Updated prediction model						
Panic symptoms: Sweating, Choking, Trembling or shaking, Fear of dying, *Shortness of breath, Dizziness, Palpitations						
Score	Sensitivity	Specificity	Classified Correctly	LR+	LR-	Youden Index
≥ 1	99.20%	31.60%	57.90%	1.451	0.0253	30.80%
≥ 2	93.60%	60.20%	73.20%	2.352	0.1063	53.80%
**≥ 3**	**78.40%**	**85.70%**	**82.90%**	**5.488**	**0.252**	**64.10%**
≥ 4	45.60%	93.30%	74.70%	6.8751	0.5826	38.90%
≥ 5	26.40%	99.50%	71.00%	51.7444	0.7398	25.90%
≥ 6	10.40%	100.00%	65.10%	-{{-}}-	0.896	10.40%
≥ 7	0.80%	100.00%	61.30%	-{{-}}-	0.992	0.80%

Items in bold indicate optimal cut-off scores.

LR+, Positive likelihood ratio; LR-, Negative likelihood ratio.

*The symptoms shortness of breath, dizziness, and palpitations overlap in both the original and updated prediction models.

In deriving the updated model, the stepwise logistic regression model identified seven symptoms as significant predictors of panic-related anxiety: shortness of breath, dizziness, palpitations, trembling or shaking, fear of dying, choking, and sweating. When tested at an optimal cutoff score of ≥3 symptoms, the updated model achieved an area under the curve (AUC) of 0.88, with a sensitivity of 78.4% and specificity of 85.7%. The Youden index was 64.1%, indicating good discriminatory ability (**Table 2**). Positive and negative likelihood ratios were 5.49 and 0.25, respectively, further supporting the model’s utility in distinguishing patients with panic-related anxiety from those without. Shortness of breath, dizziness, and palpitations were significant predictors for panic-related anxiety in both the original and updated models, but the remaining items did not overlap (See **Table 2**).

## 4 Discussion

In our initial model development study, the original prediction model showed excellent separation between those with and without panic-related anxiety at a cutoff score of ≥ 3. In this derivation of an updated model, the performance of the original model decreased, possibly due to differences in the patient population of the validation sample. The new model’s AUC and diagnostic performance were superior, validating the updated symptom combination as a more accurate tool for detecting panic-related anxiety in the ED setting. These improved metrics indicate that the updated model has good discrimination and a clinically useful balance between sensitivity and specificity for ED screening, with an optimal cutoff score of ≥ 3. It is well-known that prediction models tend to perform better on data on which the model was constructed than on new data (14). Therefore, our findings underline the importance of ongoing evaluation and refinement of predictive models to maintain their accuracy and generalizability across different settings.

### 4.1 Clinical implications

Consistent with previous studies (6, 8, 9), nearly 40% of participants in the present study met diagnostic critiera for panic-related anxiety and physician detection was low in the ED setting. Due to the physical nature of panic attack symptoms, it is not uncommon for patients to initially seek care in medical settings (4, 15, 16), which may lead to considerable delays in receiving appropriate diagnosis and treatment. For example, data from a worldwide meta-analysis found a peak age of onset of 15.5 years for first panic attack symptoms, in contrast to a peak age at first diagnosis of panic disorder of 39.5 years (17). More than 60% of patients who visited medical settings following their first panic attack reported that these visits did little to address their concerns and may have served to increase their anxiety about future attacks (16).

When patients with panic-related anxiety do not receive timely information regarding the cause of their physical symptoms, they are more likely to return to the ED when symptoms re-occur (18, 19). This pattern has been observed worldwide and remains a considerable public health problem leading to poor long-term patient outcomes, repeat ED admissions, and unnecessary medical costs (4, 20–22). Systematic screening for panic-related anxiety by ED clinicians is likely to help considerably with early detection and right-siting of these patients earlier in the course of illness. In practice, this brief symptom-based model may support ED screening by providing clinicians with a rapid, structured way to identify patients at higher likelihood of panic-related anxiety, facilitating timely reassurance and appropriate referral after medical causes are excluded. Given its brevity and symptom-based format, the SCID could feasibly be administered by triage nurses, emergency physicians, or other trained ED staff as part of routine assessment.

### 4.2 Strengths and limitations

Strengths of the present study include a relatively large sample size, the use of gold-standard diagnostic interview assessments of panic-related anxiety, and the availability of complete data for all participants. Limitations include recruitment from a single ED, over-representation of Indian participants compared to the Singapore general population, and exclusion of a number of potential participants due to language barriers. Furthermore, screening of participants was restricted to weekday daytime hours, which may have affected the representativeness of the sample. Although the SCID has been widely used in multilingual clinical settings, the Mandarin administration used in this study has not undergone independent psychometric validation within this sample, which may have introduced measurement variability. Furthermore, some panic-like symptoms may have been substance-induced (e.g., related to caffeine, stimulants, or medications) rather than attributable to panic disorder, which could have contributed to misclassification and influenced model performance.

## 5 Conclusions

Panic-related anxiety should be strongly considered in ED patients with cardiopulmonary complaints, who frequently present to emergency medicine and are often challenging to diagnose. We have externally validated and updated a brief clinical multivariable diagnostic prediction model for timely screening of panic-related anxiety in ED patients, particularly those with chest pain who also endorse shortness of breath, dizziness, and palpitations which are the most prevalent physical symptoms in panic attacks (4). Implementing such an interview may improve early detection and proper management of patients with panic-related anxiety at presentation to the ED. Future research is needed to more fully evaluate the model’s clinical utility and impact on physician decision-making.

## Data Availability

The raw data supporting the conclusions of this article will be made available by the authors, without undue reservation.

## References

[1] Davidoff J Christensen S Khalili DN Nguyen J Ishak WW . Quality of life in panic disorder: looking beyond symptom remission. Qual Life Res. (2012) 21:945–59. doi: 10.1007/s11136-011-0020-7, PMID: 21935739

[2] Roy-Byrne PP Wagner AW Schraufnagel TJ . Understanding and treating panic disorder in the primary care setting. J Clin Psychiatry. (2005) 66 Suppl 4:16–22., PMID: 15842183

[3] Deacon B Lickel J Abramowitz JS . Medical utilization across the anxiety disorders. J Anxiety Disord. (2008) 22:344–50. doi: 10.1016/j.janxdis.2007.03.004, PMID: 17420113

[4] Tunnell NC Corner SE Roque AD Kroll JL Ritz T Meuret AE . Biobehavioral approach to distinguishing panic symptoms from medical illness. Front Psychiatry. (2024) 15:1296569. doi: 10.3389/fpsyt.2024.1296569, PMID: 38779550 PMC11109415

[5] Buccelletti F Ojetti V Merra G Carroccia A Marsiliani D Mangiola F . Recurrent use of the Emergency Department in patients with anxiety disorder. Eur Rev Med Pharmacol Sci. (2013) 17 Suppl 1:100–6., PMID: 23436671

[6] Foldes-Busque G Denis I Poitras J Fleet RP Archambault P Dionne CE . A closer look at the relationships between panic attacks, emergency department visits and non-cardiac chest pain. J Health Psychol. (2019) 24:717–25. doi: 10.1177/1359105316683785, PMID: 28810369

[7] Zane RD McAfee AT Sherburne S Billeter G Barsky A . Panic disorder and emergency services utilization. Acad Emerg Med. (2003) 10:1065–9. doi: 10.1197/S1069-6563(03)00349-X, PMID: 14525739

[8] Sung SC Rush AJ Earnest A Lim LEC Pek MPP Choi JMF . A brief interview to detect panic attacks and panic disorder in emergency department patients with cardiopulmonary complaints. J Psychiatr Pract. (2018) 24:32–44. doi: 10.1097/PRA.0000000000000283, PMID: 29320381

[9] Foldes-Busque G Marchand A Chauny JM Poitras J Diodati J Denis I . Unexplained chest pain in the ED: could it be panic? Am J Emerg Med. (2011) 29:743–51. doi: 10.1016/j.ajem.2010.02.021, PMID: 20825891

[10] Department of Statistics . Census of Population 2020 Statistical Release 1: Demographic Characteristics, Education, Language and Religion 2020. Singapore: Ministry of Trade & Industry, Republic of Singapore. (2020).

[11] Fong RY Glen WSS Mohamed Jamil AK Tam WWS Kowitlawakul Y . Comparison of the Emergency Severity Index versus the Patient Acuity Category Scale in an emergency setting. Int Emerg Nurs. (2018) 41:13–8. doi: 10.1016/j.ienj.2018.05.001, PMID: 29887281

[12] First MB Spitzer RL Gibbon M Williams JBW . Structured clinical interview for DSM-IV-TR axis I disorders, research version, patient edition. (SCID-I/P). New York: Biometrics Research, New York State Psychiatric Institute (2002).

[13] Foldes-Busque G Fleet RP Denis I Poitras J Chauny JM Diodati JG . Nonfearful panic attacks in patients with noncardiac chest pain. Psychosomatics. (2015) 56:513–20. doi: 10.1016/j.psym.2014.07.005, PMID: 25583556

[14] Bleeker SE Moll HA Steyerberg EW Donders AR Derksen-Lubse G Grobbee DE . External validation is necessary in prediction research: a clinical example. J Clin Epidemiol. (2003) 56:826–32. doi: 10.1016/S0895-4356(03)00207-5, PMID: 14505766

[15] Katerndahl DA Realini JP . Where do panic attack sufferers seek care? J Fam Pract. (1995) 40:237–43., PMID: 7876780

[16] Pane-Farre CA Stender JP Fenske K Deckert J Reif A John U . The phenomenology of the first panic attack in clinical and community-based samples. J Anxiety Disord. (2014) 28:522–9. doi: 10.1016/j.janxdis.2014.05.009, PMID: 24973697

[17] Solmi M Radua J Olivola M Croce E Soardo L Salazar de Pablo G . Age at onset of mental disorders worldwide: large-scale meta-analysis of 192 epidemiological studies. Mol Psychiatry. (2022) 27:281–95. doi: 10.1038/s41380-021-01161-7, PMID: 34079068 PMC8960395

[18] Heppell JL Denis I Turcotte S Fleet RP Dionne CE Foldes-Busque G . Incidence of panic disorder in patients with non-cardiac chest pain and panic attacks. J Health Psychol. (2021) 26:985–94. doi: 10.1177/1359105319859062, PMID: 31250658

[19] Foldes-Busque G de Lafontaine MF Turcotte S Denis I . Are patients at risk for developing panic disorder after an emergency department visit with noncardiac chest pain? J Acad Consult Liaison Psychiatry. (2022) 63:23–31. doi: 10.1016/j.jaclp.2021.07.011, PMID: 34352451

[20] Musey PI Lee JA Hall CA Kline JA . Anxiety about anxiety: a survey of emergency department provider beliefs and practices regarding anxiety-associated low risk chest pain. BMC Emerg Med. (2018) 18:10. doi: 10.1186/s12873-018-0161-x, PMID: 29540151 PMC5853064

[21] Coley KC Saul MI Seybert AL . Economic burden of not recognizing panic disorder in the emergency department. J Emerg Med. (2009) 36:3–7. doi: 10.1016/j.jemermed.2007.06.002, PMID: 17933481

[22] Bandelow B Michaelis S . Epidemiology of anxiety disorders in the 21st century. Dialogues Clin Neurosci. (2015) 17:327–35. doi: 10.31887/DCNS.2015.17.3/bbandelow, PMID: 26487813 PMC4610617

